# Engagement Strategies for Self-Monitoring Symptoms of Bipolar Disorder With Mobile and Wearable Technology: Protocol for a Randomized Controlled Trial

**DOI:** 10.2196/resprot.9899

**Published:** 2018-05-10

**Authors:** Amy Cochran, Livia Belman-Wells, Melvin McInnis

**Affiliations:** ^1^ Department of Biostatistics and Medical Informatics University of Wisconsin-Madison Madison, WI United States; ^2^ Brown University Providence, RI United States; ^3^ Department of Psychiatry University of Michigan - Ann Arbor Ann Arbor, MI United States

**Keywords:** bipolar disorder, self-management, patient participation, mobile applications, wearable electronic devices

## Abstract

**Background:**

Monitoring signs and symptoms in bipolar disorder (BP) is typically based on regular assessments from patient-clinician interactions. Mobile and wearable technology promises to make monitoring symptoms in BP easier, but little is known about how best to engage individuals with BP in monitoring symptoms.

**Objective:**

The objective of this study was to provide the rationale and protocol for a randomized controlled trial that investigates engagement strategies for monitoring symptoms of BP, including the strategies of using activity trackers compared with self-reports and reviewing recorded symptoms weekly with an interviewer.

**Methods:**

A total of 50 individuals with BP will be recruited from the Prechter Longitudinal Study of Bipolar Disorder at the University of Michigan to participate in a 6-week study. Participants will monitor their symptoms through an activity tracker (Fitbit Alta HR) and a mobile phone app designed for this study. In addition to monitoring symptoms, participants have a 50-50 chance of being assigned to an arm that reviews self-reports and activity information weekly. Statistical tests will be performed to test hypotheses that participants adhere to activity tracking significantly more than self-reporting, prefer activity tracking significantly more than self-reporting, and better adhere to both activity tracking and self-reporting when reviewing collected information weekly.

**Results:**

Recruitment commenced in November 2017. The first group of participants began the study in January 2018.

**Conclusions:**

This study aims to establish strategies to engage individuals with BP in monitoring their symptoms with mobile and wearable technology. Better engagement strategies are expected to aid current efforts in bipolar research and clinical care, from the development of new mobile phone apps to providing the right intervention to the right individual at the right moment.

**Trial Registration:**

ClinicalTrials.gov NCT03358238; https://clinicaltrials.gov/ct2/show/NCT03358238 (Archived by WebCite at http://www.webcitation.org/6yebuNfz5)

**Registered Report Identifier:**

RR1-10.2196/9899

## Introduction

### Background

Bipolar disorder (BP) is a chronic illness of pathological shifts in mood ranging from mania to depression. Management of BP is typically a combination of medication with psychosocial therapy; however, relapse is common, resulting in overall inadequate care in practice and over the course of the illness [1]. With limitations in treatment options, along with imprecise guidelines on when, where, and how to intervene, promising psychosocial therapies require adaptive strategies to better address the specific needs of individuals in a timely manner [2]. To accomplish this requires evidence-based practices for adapting a psychosocial therapy, for which mobile health and wearable technology play a key role [3].

Effective adaptive interventions hinge on collecting momentary and pertinent observations. For BP, observations would ideally be collected as frequent as twice daily and for as long as weeks or years to capture the following characteristic features of BP: diurnal patterns [4], rapid mood shifts [5], full-length manic and depressive episodes [6], and waiting periods before medications take effect [7]. Monitoring symptoms in BP is also important, irrespective of the need for adaptive interventions. The World Health Organization (WHO) recommends that individuals with BP monitor their symptoms, and psychosocial therapies for BP often require individuals to monitor their symptoms [8].

Historically, symptoms in BP are monitored through self-reporting, often with mood on a one-dimensional scale such as the Mood 24/7 scale [9] and the National Institute of Mental Health (NIMH) Life Chart Method [10]. Mood, however, may not be one-dimensional, as manic and depressive symptoms frequently appear together [11]. NIMH’s Research Domain Criteria [12], for example, considers a negative valence domain separately from a positive valence domain, allowing for the possibility that each valence domain is regulated separately. Self-reports also depend on an individual’s mood and time of day [4] and require individuals to actively record symptoms, a burden that leads to disengagement [13,14]. In sum, self-reports are subjective and time-intensive and lose information, which may limit their utility in charting BP.

Increasingly, BP symptoms are being monitored *passively* through sensors on mobile phones and wearable devices [15-21]. Mobile phone platforms MONitoring, treAtment and pRediCtion of bipolAr Disorder Episodes (MONARCA) [22] and Predicting Individual Outcomes for Rapid Intervention (PRIORI) [23] predict mood from patterns of speech and behavior from recorded calls, number of phone calls, and phone call duration. Wearable devices such as activity trackers monitor physical activity, as well as circadian and sleep rhythms. Circadian and sleep rhythms are regulated in therapies such as sleep deprivation and interpersonal and social rhythm therapy [24,25]. Circadian rhythms are thought to be central to BP etiology, with connections to risk genes, animal models, and pharmacological therapy [26], and actigraphic variables are linked to genetic differences between individuals with BP-I and without BP-I that include later wake times, longer sleep durations, and lower activity levels in BP-I subjects during euthymia [27]. Data from mobile phone and wearable devices can also be fitted to models of circadian and sleep rhythms [28], which have provided insight into normal sleep habits around the world [29].

### Objectives

The benefits and needs are clear for monitoring symptoms of BP using mobile and wearable technology, but we still do not know how best to engage individuals with BP in this task. In this paper, we describe the protocol for a study to answer this question (ClinicalTrials.gov NCT03358238). Briefly, individuals with BP will interact with a mobile phone app and activity tracker over 6 weeks. They will report their symptoms twice-daily with the mobile phone app while activity, sleep, and heart rate are recorded with an activity tracker. The study implements 3 engagement strategies: using activity trackers rather than self-reports; reviewing recorded symptoms with another person on a weekly basis; and synthesizing a person’s data into charts and graphs. We hypothesize that individuals with BP will prefer and better adhere to monitoring their symptoms when using activity trackers over self-reports and when reviewing their recorded symptoms weekly with an interviewer.

## Methods

### Recruitment

In total, 50 individuals with BP will be recruited from the Prechter Longitudinal Study of Bipolar Disorder [30] to participate in a 6-week, 2-arm randomized controlled trial. This 6-week study has been approved by institutional review boards at the University of Michigan (HUM126732) and University of Wisconsin (2017-1322). Participants in the Prechter study have already completed a Diagnostic Interview for Genetic Studies about their health and mental illness history. We will access the diagnostic information and other data from the longitudinal study so as not to repeat the interview process. Individuals will be included if they (1) have agreed to be contacted for future research, (2) have a mobile phone, and (3) have a diagnosis of BP. Participation is open to men and women; to adults (aged 18 years or older); and to all ethnic and racial groups. To select participants from the larger trial, a data manager pulled a query of participants with diagnoses of either BP I, BP II, or BP NOS (not otherwise specified) and who have authorized contact for future studies. Participants are ordered by authorization date, with most recent dates at the top of the list and recruitment emails are sent in this order. Individuals who express interest and have a mobile phone are then consented.

The study aims to be balanced by age and gender and to achieve adequate representation of minorities as reflected by the diversity of Washtenaw and surrounding Counties of Michigan, in which most participants live. All participants will be consented, which involves a discussion over the phone, followed by submission of consent electronically through the data capture software REDCap (see Multimedia Appendix 1 for Informed Consent document). Sample size and observation length are based on 3 auxiliary studies of the Prechter study finding significant associations between variables and significant differences in measurements between two groups, assuming about 10% to 20% attrition.

### Randomization

Each participant is randomly assigned to one of the two arms (Arm NR=No Review or Arm R=Review), stratified by age and gender. Each participant has a 50-50 chance of being assigned to Arm R. Following weekly mood assessments, Arm R reviews information collected over the week with an interviewer, whereas Arm NR does not discuss this information. Subjects and interviewers will necessarily be unblinded. A randomized list of assignments to one of the two arms was generated for 4 groups: women <40 years of age (N=13), women ≥40 years of age (N=13), men <40 years of age (N=12), and men ≥40 years of age (N=12). The randomized list was generated using a random number generator in Matlab (Mathworks; Natick, MA) and a block design, which involves generating random assignments in smaller blocks of participants and then randomly permuting the order of blocks. Because the 2 women groups had an odd number, these blocks were each unbalanced but together ensured equal participants in each arm; the arm that was over-represented in each group was chosen uniformly at random. Additional randomized blocks were appended to the randomization list in case of dropout. Although unblinded, arm assignments are not revealed until individuals are recruited and consented to avoid selection bias.

### Study Design

A summary of participation in the study is given in Table 1. Participation involves 7 weekly phone interviews and the use of mobile and wearable technology over 6 weeks. Each participant wears a Fitbit Alta HR to collect information about physical activity, sleep, and heart rate. They also download a mobile phone app designed specifically for this study called Lorevimo, which we will discuss in detail below. Briefly, the study app Lorevimo prompts the user twice-a-day (once in the morning and once in the evening) on questions about manic and depressive symptoms and medication adherence. The mobile phone app also allows the participant to review and chart information collected from self-reported app survey and activity tracker and from data processed with computational algorithms. Participants are compensated for each phone interview but not using the app or activity tracker (see Multimedia Appendix 2 for detailed protocol).

The first phone interview marks the start of the study for each participant. The interviewer conducts 3 surveys over the phone to assess mood and general health. Manic symptoms are assessed with a Young Mania Rating Scale (YMRS) [31]; depressive symptoms are assessed with a Structured Interview Guide for the Hamilton Rating Scale of Depression (SIGHD) [32]; and general health is assessed using the 36-Item Short Form Health Survey (SF-36) developed by RAND [33]. After the first phone interview, participants are contacted by phone at weekly interviews. The next 5 interviews will also entail an assessment of manic and depressive symptoms using the YMRS and SIGHD. In addition to these assessments, participants in Arm R will also review with the study team what they reported over the week about mood and medication adherence, and what the activity tracker reported about their sleep, heart rate, and physical activity. The seventh and final phone interview marks the end of the 6-week study. At this interview, the interviewer again conducts 3 surveys used at the start of the study: YMRS, SIGHD, and SF-36. In addition, the interviewer also conducts a final survey designed specifically for the study called the Engagement Assessment (available in the Multimedia Appendix 3).

### Design of Study App

**Figure 1:**
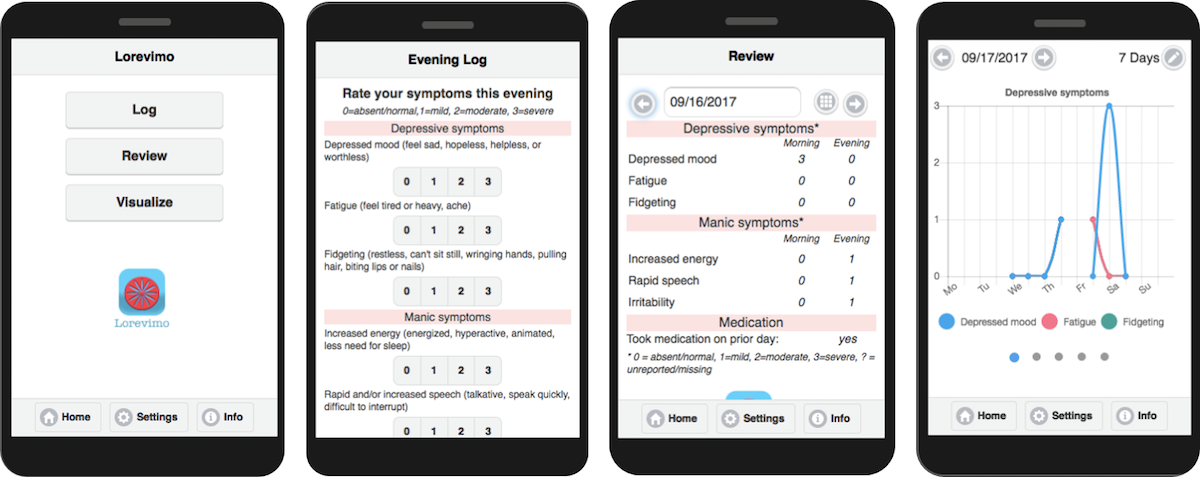
Screenshots of study app Lorevimo, which derives its name from the app’s 3 main functions: to log, review, and visualize your mood.

We designed an app, called Lorevimo, for monitoring symptoms in BP. Its name derives from the 3 main functions of the app: Log, Review, and Visualize your Mood (Figure 1). We focused on developing a *simple* app with minimal components, so results from this study could generalize to other tracking apps in BP (which are numerous) [34]. At a minimum, we wanted the app to allow users to log their symptoms and receive reminders to log their symptoms. We then added 2 functionalities—reviewing, and visualizing their logged symptoms—to encourage app usage, which we discuss in detail below.

Lorevimo is currently restricted to participants by requiring a coded username and password provided to each participant upon entering the study. Participants can find the free app for Android through Google Play and for iPhone through iTunes. If participants are away from their phone, they can also access the app through any standard Web browser. However, push notifications are not available for the Web-based app. The app was developed using the online software Appery.io (Appery LLC; Walnut Creek, CA), which combines drag-and-drop functionality with Javascript to flexibly allow for quick development and advance control of development. Appery.io also provides each app with backend servers, database management, application programming interface integration, push notifications, and automatic packaging for each app into Android, iPhone, and Web-based apps.

#### Log Your Mood

Lorevimo’s main function is to log manic and depressive symptoms and medication adherence (Figure 1). Users can log symptoms, once in the morning and once in the evening, to capture diurnal patterns in mood characteristic of BP. Morning and evening is defined based on a participant’s self-reported wake and bed times on weekdays and weekends: respectively, the 6-hour window spanning 2 hours before to 4 hours after their typical wake time and the 6-hour window spanning 4 hours before to 2 hours after their typical bed time. Typical bedtimes or wake times are entered by the user when they first log into the app and can be changed anytime under Settings in the app. Automatic reminders via push notifications are sent at 2-hour intervals for individuals who are yet to log symptoms, are within the appropriate window, and not too close to their typical bedtime or wake time.

**Table 1:** Summary of study participation and information collected on each participant.

Interaction	Instruments	Study days
Entrance phone interview	YMRS^a^, SIGHD^b^, RAND SF-36^c^	0
App self-report	3 items from YMRS, 3 items from SIGHD, medication adherence (only in the morning)	0-42, twice
Offline computed variables	Computed variables: circadian phase and amplitude and cumulative sleep debt	0-42
Activity tracker	Activity, sleep, heart rate	0-42
Weekly phone interview	Both arms: YMRS, SIGHD. Arm R only: review of collected data from app and activity tracker	7, 14, 21, 28, 35
Exit phone interview	YMRS, SIGHD, RAND SF-36, Engagement survey	42

^a^YMRS: Young Mania Rating Scale.

^b^SIGHD: Structured Interview Guide for the Hamilton Rating Scale of Depression.

^c^SF-36: 36-Item Short Form Health Survey.

Six symptoms are logged each morning and evening: 3 for mania (increased energy, rapid speech, irritability) and 3 for depression (depressed mood, fidgeting, fatigue). In the morning, users also mark whether they took their medicine on the previous day. We remark that the 6 symptoms capture the various ways in which symptoms manifest in BP, including *anxious* depression (depressed mood+fidgeting); *anehedonic* depression (depressed mood+fatigue); *euphoric* mania (increased energy+rapid speech); *irritable* mania (irritability); and *mixed* states (depressed mood+increased energy). All symptoms are scored on a 0 to 3 scale with 0=absent/normal, 1=mild, 2=moderate, and 3=severe. Ordinal scales were used as opposed to visual analog scales to be consistent with YMRS and SIGHD to clearly identify the absence of symptoms as zero and to ensure individuals can discriminate between choices.

In choosing these 6 symptoms, our goal was to maximize the consistency between the logged symptoms via the app and our *gold standard* or *ground truth* for manic and depressive severity, without overburdening the user with long surveys. Our ground truth was total scores on the interview-based YMRS and SIGHD. We thus focused on logging 6 symptoms chosen from the YMRS and SIGHD. These 6 symptoms were identified from a preliminary analysis of longitudinal surveys of YMRS and SIGHD from 27 individuals with either BP I or BP II from the Prechter Longitudinal Study of Bipolar Disorder at the University of Michigan. Surveys were performed weekly, for a total number of weeks per individual ranging from 2 to 52 weeks, with a median of 20 weeks. Using this data, we performed an exhaustive search of all 6-item sets chosen from the 11-item YMRS and 17-item SIGHD. For each 6-item set, we built linear models, one to predict total YMRS score and one to predict total SIGHD score, from the corresponding responses for the 6 items and measured mean-squared error in each linear model. Samples were weighted inversely by the number of samples per person, to ensure individuals contributed equally to estimation. We then identified which 6-item set (of over 300,000 possible sets) had the smallest sum of mean-squared error for predicting YRMS scores and SIGHD score, which resulted in the aforementioned 6 symptoms. The resulting 6-item set has not been validated yet for use in a mobile app.

#### Review and Visualize Your Mood

**Figure 2:**
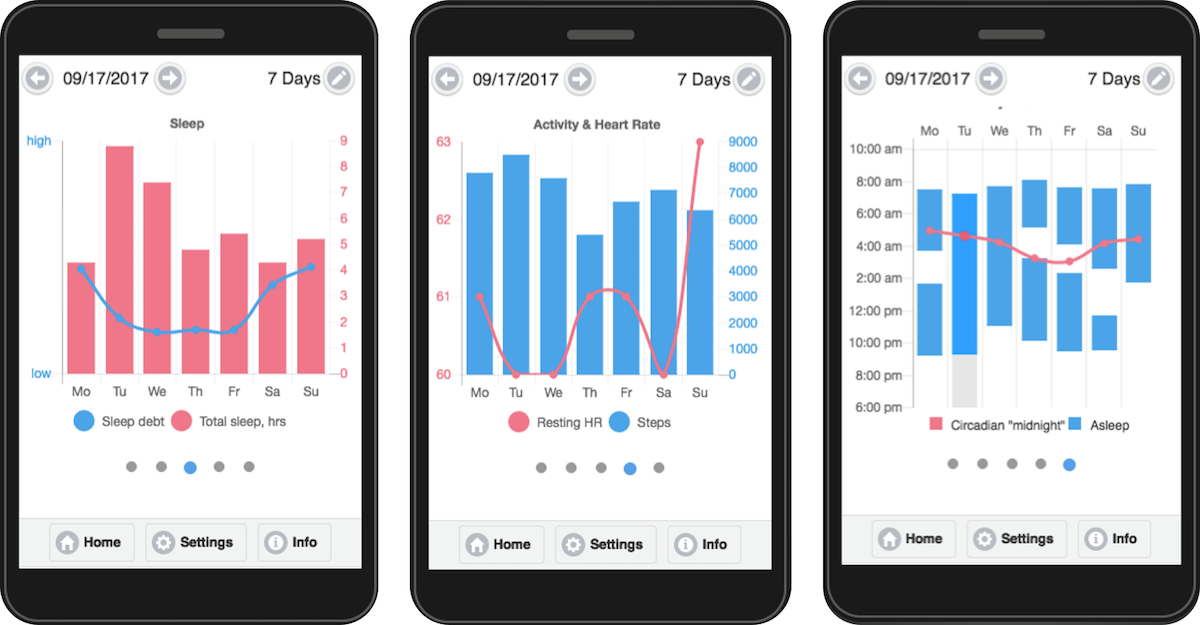
Screenshots of Visualize portion of study app showing data collected from Fitbit and variables computed from mathematical modeling. HR: heart rate.

The remaining 2 functions of the app are to provide users a place to *review* their reported symptoms and *visualize* their symptoms and Fitbit data with charts and graphs (Figures 1 and 2). Upon clicking Review from the app’s home page, the user is shown a report on what they reported for the current day through the app, which consists of at most 6 morning symptoms, 6 evening symptoms, and whether they took their medication on the prior day. Missing items are left blank. The user can then navigate to view reports from other days. Upon clicking Visualize from the app’s home page, the user is shown a graph of their depressive symptoms over the past week. They can change the time period’s length (3, 7, or 28 days) and ending day. Upon swiping left, they can also view manic symptoms, sleep patterns, activity and heart rate patterns, and daily circadian and sleep rhythms.

These functions were designed to serve dual roles: (1) to help an individual with BP manage their disorder and (2) to encourage individuals to continue to log their symptoms with the app. These roles may be accomplished in several ways. First, a participant who reviews/visualizes their symptoms should have increased awareness of symptoms, a cornerstone of many effective psychosocial therapies [2]. Second, the participant could gain insight into their illness, for example, helping them to recognize that when they exercise, their mood improves. Third, they could be warned about an impending manic or depressive episode, as they see their symptoms begin to reach severe levels. Meanwhile, if these functions indeed help a user manage their disorder, then they may be more greatly motivated to log their symptoms, which is ultimately the purpose of this study. Even if these functions do not help with BP management, individuals may still be motivated to log their symptoms and use their activity tracker, knowing that they will gain *information* from this transaction. Information-seeking is believed to be a mechanism by which individuals engage with mobile and wearable devices.

### Activity Tracking

We give each participant a Fitbit Alta HR to track activity, sleep, and heart rate for the duration of the study. They are provided the original instructions that accompany the Fitbit for setting up the device. We do not provide additional instructions for setting up the Fitbit but do instruct them on how to provide us access to their Fitbit data and ask them to wear the Fitbit except to shower or charge the device. While mobile phones are capable of tracking activity and would be less intrusive as a wearable device thereby helping to reduce stigma, we opted for a wearable device to be able to track sleep and heart rate in addition to activity and to avoid differences in how activity is measured resulting from different mobile phone devices or different habits in how mobile phone devices are carried. Sleep and exercise may be important to track, as they are considered part of a healthy lifestyle and believed to be just as important in BP [8]. Furthermore, many clinical assessments of BP use measures related to sleep and activity, such as insomnia, psychomotor retardation, and energy levels [31,32]. Mania, for example, is marked by less need for sleep and increased activity, whereas depression is marked by decreased activity and hypersomnia. Heart rate may also be important to track in BP, since heart rate properties may reflect increased stress [35], a trigger of manic and depressive symptoms [36]. In sum, daily activity, sleep, and heart rate could be important markers of mood and targets for therapy.

Participants are asked to set-up their own Fitbit account and sync a Fitbit Alta HR to their own account. We then ask each participant to provide us access to their Fitbit data on activity, sleep, and heart rate. We store the access and refresh tokens granted to us and link these tokens to the participant’s coded study username on a server. This server than queries Fitbit server once a day to collect daily information from participant’s Fitbit. Collected activity information consists of steps, minutes active, minutes sedentary, distance traveled, calories when active, and total calories. Collected sleep information consists of sleep periods with duration; efficiency; start and end times; time in bed; levels; and minutes asleep, awake, to fall asleep, and after wake time. Finally, collected heart rate information consists of resting heart rate; and minimum heart rate, maximum heart rate, and duration in 4 different heart rate zones. Much of this information is relayed to the study app Lorevimo for participants to visualize over time.

### Mathematical Modeling of Sleep and Circadian Patterns

Circadian rhythms are potentially important markers of BP but are not tracked by Fitbit. To that end, we model circadian rhythms using the Forger-Jewett-Kronauer model [28]. Light levels are usually inputted into the model to reflect its ability to shift circadian phase but since light is not measured by a Fitbit, we assume an individual is exposed to 150 lux when awake and 0 lux when asleep [29,37], whereas this latter information is available from the Fitbit. A homeostat component, another factor in sleep drive, will also be modeled such that it increases while awake and decays while asleep [38]. These models yield 4 markers: circadian phase, circadian amplitude, maximum homeostat, and minimum homeostat. These models are processed by a server with information relayed to the study app Lorevimo. A participant can view their circadian phase and maximum homeostat, which are respectively renamed *circadian midnight* and *sleep debt* for the app, through charts and graphs under the Visualize feature of the app.

### Engagement Survey

We designed an *engagement survey* which will be administered over the phone at the end of the study (Multimedia Appendix 3). This survey consists of 17-items, each aimed at understanding how best to engage individuals with BP in monitoring symptoms with a mobile phone app and activity tracker. The engagement survey asks questions about their preference toward using a mobile phone app versus activity tracker, general feelings and attitudes toward using a mobile phone app, activity tracker, and charts/graphs for monitoring symptoms and toward reviewing information weekly with another person. Additional items ask participants to identify top 3 symptoms or patterns to monitor, top 3 barriers to monitoring symptoms, and top 3 uses for monitoring symptoms.

### Data Analysis

#### Primary Outcome Measures

We identified four primary outcome measures for evaluating how best to engage BP individuals in monitoring their symptoms with mobile and wearable technology. Specifically, our primary outcome measures test two engagement strategies for monitoring symptoms in BP: using activity trackers over mobile phone and reviewing data collected weekly with an interviewer. The former strategy is evaluated with the first and second primary outcomes: *proportion of participants who report they are more likely to use a* mobile phone *app over an activity tracker to monitor their symptoms* and *proportion of participants who have higher adherence rates for self-reporting symptoms than adherence rates for activity tracking.* Likelihood of using an app over an activity tracker is measured using the engagement survey at study end. The relevant question asks, “Which are you more likely to use to monitor your symptoms” and has 2 mutually exclusive options for an answer: “An activity tracker” or “A mobile phone app.” This outcome directly tests the hypothesis that BP individuals prefer activity trackers over mobile phone apps for monitoring their symptoms. Adherence rate for activity tracking is measured as the proportion of study days with at least 12 hours of activity tracking, which is determined indirectly using total minutes Fitbit measures heart rate. Adherence rate for self-reporting symptoms is measured as the proportion of study days with at least 50% of daily self-reports survey questions completed.

The second and third outcome tests the hypothesis that reviewing collected data weekly will increase engagement in monitoring symptoms. The second outcome is *difference in adherence rates for self-reporting symptoms between individuals who review their data weekly with an interviewer (Arm R) compared with individuals who do not review their data weekly with an interviewer (Arm NR).* The third outcome is *difference in adherence rates for activity tracking between individuals who review their data weekly with an interviewer (Arm R) compared with individuals who do not review their data weekly with an interviewer (Arm NR).*

To accompany these measures of adherence and engagement, we will also use other responses on the engagement survey to further examine reasons why an individual might monitor their symptoms with either a mobile phone or an activity tracker. Since responses are in the form of multiple-choice options, we will report number and proportion of participants in the entire group and in each arm, who choose each option.

#### Secondary Outcome Measures

Secondary outcome measures evaluate whether symptoms improve in the study: change from baseline in severity of manic symptoms, as measured with the YMRS; and change from baseline in severity of depressive symptoms, as measured with the 17-item SIGHD. We hypothesize that by tracking symptoms throughout the study, individuals with BP improve their symptoms. Establishing validity of this hypothesis will serve to reinforce the importance of monitoring symptoms in BP as a target in therapy.

#### Exploratory Aims

Along with evaluating primary and secondary measures above, the data collected from this study provides opportunities to examine other questions. For one, we want to establish validity of our mobile-based 6-item survey for tracking BP, by determining to what extent the 6 symptoms can explain total scores on the weekly interview-based YMRS and SIGHD total scores as was demonstrated in our preliminary analysis. Additionally, we want to examine other strategies for increasing engagement in monitoring BP symptoms. To that end, we will examine responses on our engagement survey about providing charts and graphs of user data over time, top 3 uses of technology for monitoring symptoms, top 3 barriers of preventing technology from being used to monitor symptoms, and top 3 symptoms or patterns to monitor. Finally, we want to evaluate mathematical models for personalizing phenotypes of BP based on *daily* patterns of mood in BP [11].

#### Statistical Tests

For the first two primary outcome measures, we will test whether proportions are statistically different from 0.5 using a binomial test. These tests answer whether users prefer and adhere to monitoring symptoms using apps over activity trackers. For the second two primary outcome measures, we will test whether adherence rates are statistically different between arms using a 2-sample Student *t*-test if variances can be assumed to be equal, otherwise a Welch *t* test will be used. These tests answer whether users better adhere to monitoring symptoms with apps and activity trackers when reviewing collected information weekly with an interviewer. For the secondary outcome measures, we will test whether individuals experience a statistically significant increase in YMRS and SIGHD scores from baseline over the course of the 6-week study using McNemar test, which compares the number of individuals whose survey scores increased versus those whose survey scores decreased. For all statistical tests, significance will be considered a *P* value less than .05.

## Results

We began recruiting for this study on November 27, 2017. As of January 1, 2018, we had 3 people enrolled and consented in the study. Study participations began in January 2018. The study app was released to Google Play and iTunes in Fall 2017 but is currently password-protected to restrict use to study participants.

## Discussion

### Principal Findings

We presented a study on engaging individuals with BP in self-monitoring symptoms with a mobile phone app and activity tracker. Individuals with BP are characterized by extreme shifts in their mood ranging from mania to depression, which often arise quickly and without warning. The WHO recommends that individuals with BP monitor their symptoms [8], and many psychosocial interventions in BP include some form of monitoring symptoms to provide early warnings, raise awareness of symptoms, and provide insight into an individual’s illness [2]. Perhaps most importantly, monitoring of symptoms is a minimum requirement to determine effective adaptive interventions that deliver the *right* psychosocial interventions to the *right* individual at the *right* moment [14]*.* Making it easier to monitor symptoms is the promise of mobile and wearable technology, mirroring a larger trend for chronic diseases such as BP.

Our goal is to ensure that such technology is optimally designed and evidence-based for monitoring symptoms in BP. We thus focus on formally testing 3 engagement strategies: using activity trackers rather than self-reports in a mobile phone app; reviewing collected information weekly; and synthesizing information with charts and graphs. We expect to verify that the individuals with BP prefer and better adhere when using activity trackers over self-reports in a mobile phone app to monitor their symptoms, and when information is reviewed weekly. We also expect that individuals with BP, in the study, will experience a decrease in manic and depressive symptoms, reinforcing the idea that monitoring symptoms can alone be therapeutic and can provide further impetus for individuals with BP to monitor their symptoms. Our study app was simple in its design: users can log, review, and visualize their mood. This simplicity ensures study results will generalize to the wealth of mobile and wearable technology developed for BP [15-20,22,23], translating into better technology for individuals with BP for monitoring symptoms.

### Limitations

We remark on two limitations of the study design. First, we do not track usage statistics other than whether a participant logs their symptoms. Specifically, we do not track whether a participant logs their symptoms through a mobile phone app or online, which would be useful in understanding how individuals monitor their symptoms. Second, we provide participants a Fitbit at the start of the study, which many have never used before. Introduction of the activity tracker may influence engagement. For some individuals, we expect engagement in the study to be higher than long-term engagement because of the novelty of the tracker. For others, we expect engagement in the study to be lower than long-term engagement because they are not familiar with the device. For example, we find that certain individuals do not sync their Fitbit on a regular basis. To help estimate long-term engagement, we thus ask in our engagement survey how long individuals would continue to use the activity tracker.

## Appendix

Multimedia Appendix 1 Informed Consent document.

Multimedia Appendix 2 Detailed protocol for the study.

Multimedia Appendix 3 Engagement survey: a 17-item survey to be administered over the phone at the end of the study and to assess engagement of participant in self-monitoring of symptoms. Items ask about general feelings and attitudes toward using mobile and wearable technology to monitor symptoms.

## Abbreviations

BP: bipolar disorder
NIMH: National Institute of Mental Health
SIGHD: Structured Interview Guide for the Hamilton Rating Scale of Depression
SF-36: 36-Item Short Form Health Survey
YMRS: Young Mania Rating Scale
