# Supplementary material for: Engagement Strategies for Self-Monitoring Symptoms of Bipolar Disorder With Mobile and Wearable Technology: Protocol for a Randomized Controlled Trial
Source: JMIR Res Protoc. 2018 May 10;7(5):e130. doi: 10.2196/resprot.9899 (PMC5968216; doi:10.2196/resprot.9899)
Supplement: Multimedia Appendix 1 [file resprot_v7i5e130_app1.pdf]

# UNIVERSITY OF MICHIGAN

# CONSENT TO BE PART OF A RESEARCH STUDY

## INFORMATION ABOUT THIS FORM

You may be eligible to take part in a research study. This form gives you important information about the study. It describes the purpose of the study, and the risks and possible benefits of participating in the study.

Please take time to review this information carefully. After you have finished, you should talk to the researchers about the study and ask them any questions you have. You may also wish to talk to others (for example, your friends, family, or other doctors) about your participation in this study. If you decide to take part in the study, you will be asked to sign this form. Before you sign this form, be sure you understand what the study is about, including the risks and possible benefits to you.

## 1. GENERAL INFORMATION ABOUT THIS STUDY AND THE RESEARCHERS

**1.1 Study title:**

Modeling Mood to Detect Effective Adaptive Interventions

**1.2 Company or agency sponsoring the study:**

National Institute of Mental Health (NIMH); National Institute of Health (NIH)

**1.3 Names, degrees, and affiliations of the researchers conducting the study:**

Principal Investigator:

Melvin McInnis, MD, Professor, Department of Psychiatry, University of Michigan

## 2. PURPOSE OF THIS STUDY

**2.1 Study purpose:**

Bipolar disorder is marked by chronic and severe changes in mood. We know symptoms can improve with regularly monitoring and maintenance of daily patterns of mood, sleep, stress, exercise, and medication. However, we do not know how to help individuals monitor their daily patterns or how to use these patterns to direct therapy to individuals when it is most needed. To answer these questions, we will study how individuals with bipolar disorder can use a smart-phone application and activity tracker to track their daily patterns.

## 3. INFORMATION ABOUT STUDY PARTICIPANTS (SUBJECTS)

Taking part in this study is completely **voluntary**. You do not have to participate if you don't want to. You may also leave the study at any time. If you leave the study before it is finished, there will be no penalty to you, and you will not lose any benefits to which you are otherwise entitled.

**3.1 Who can take part in this study?**

Participants with bipolar disorder who are part of the Prechter Longitudinal Study of Bipolar Disorder may be able to take part in this study.

**3.2 How many people (subjects) are expected to take part in this study?**

We are looking for 50 individuals with bipolar disorder.

## 4. INFORMATION ABOUT STUDY PARTICIPATION

### 4.1 What will happen to me in this study?

Since you are already a participant in the Longitudinal Study of Bipolar Disorder (study number: HUM606), you have already completed a Diagnostic Interview for Genetic Studies (DIGS). The DIGS information is about your health and mental illness history. If you decide to take part this study, we will access the diagnostic information and other data from the longitudinal study. This way, we will not need to repeat the interview process.

If you decide to join this study, we will mail you an activity tracker such as an Apple Watch or FitBit, along with a return box with postage paid. Once you receive the activity tracker, we will schedule a time to talk to you over the phone to assess your functioning in different areas of your life. We'll ask you to complete questionnaires and mood assessments and will explain to you how to use the study smartphone application and activity tracker, including how to report and review your information and set preferences for notifications. This will take approximately one hour.

We will ask that you wear the activity tracker at all times during the six weeks of the study, except to charge the tracker or to shower. The tracker will automatically collect information about physical activity and sleep. We will also ask you to complete questionnaires about your mood, stress, and medication adherence twice a day. These questions will be prompted automatically by the smart-phone app. We may also contact you to remind you.

You will also be contacted weekly, for the full six weeks, by the study team to complete questionnaires to assess the severity of depressive symptoms and manic symptoms. There is a one-in-two chance that in addition to these assessments you will also review with the study team what you reported over the week about your mood, stress, and medication adherence, and what the activity tracker reported about your sleep and physical activity.

At the end of the study, we will call you to assess your functioning and mood and ask you to complete questionnaires like we did at the beginning of the study. This assessment will take about one hour. You will then be asked to mail us any study equipment (activity tracker or iPad) using the return box.

Your data will be stored indefinitely with collaborators located at the University of Michigan as part of the Heinz C. Prechter Bipolar Genetics Repository (HUM10454). This data will be stored for additional future research.

Please note that we will not be actively monitoring the data collection system. If you feel that you are in crisis situation, call 911 or UMHS Psychiatric Emergency Services (PES): 734-936-5900. As a subject participating in this research study, you have certain responsibilities that may apply to this study, such as ensuring that you can reached by phone at all of your scheduled appointments, take your study medication as directed, and report any adverse reactions you may have during the study.

### 4.2 How much of my time will be needed to take part in this study?

The initial assessment and the final assessment will each take approximately one hour for a total of two hours. The daily questionnaires will each take approximately 10 minutes each day (5 minutes for a mid-morning questionnaire and 5 minutes for an evening questionnaire) for six weeks. In addition, you will be contacted by the study team each week to complete questionnaires. These will take about 20 minutes/week. You will also have a one-in-two chance of reviewing your daily questionnaires and daily activity each week after you complete the questionnaires. This review will take an additional 20 minutes/week.

### 4.3 When will my participation in the study be over?

The total length of this study is six weeks. Your participation in this study is over after you have completed the final assessment over the phone.

## 5. INFORMATION ABOUT RISKS AND BENEFITS

### 5.1 What risks will I face by taking part in the study? What will the researchers do to protect me against these risks?

The known and expected risks are:

There is a risk that personal information about you, taken from the interviews or from your cell phone and transmitted via cellphone, may be revealed to people not authorized to see this information.

Some of the questions about psychiatric symptoms may make you feel uncomfortable. As with these, and all, questions in the interview, you may decline to respond. You may also lose or misplace your cell phone and someone may find it and attempt to learn the content of your recordings.

The researchers will try to minimize these risks by:

1. We will use a secure, HIPAA-compliant, web-based application known as REDCap, along with fully automated encryption technology and secure websites (https), for data capture to minimize the risk of security breach to the files. REDCap is managed by the Michigan Institute for Clinical and Health Research at the University of Michigan.
2. Data from REDCap will be downloaded through secure internet connections and stored in secure, restricted access servers that are administered by the University of Michigan School of Information's highly trained information technology department. The servers are behind a firewall, under intrusion monitoring, and will be made accessible only to the members of the study team.
3. Data downloaded from REDCap will have all personally-identifiable information (e.g., participants' names, email addresses) removed and will be coded with a participant ID. Coded data will be stored separately from the link between the participant's personally-identifiable information and participant ID.
4. It will not be apparent to the average casual user of the cell phone that the software installed is collecting information such as physical activity and mood. When you complete the questionnaires, you will be prompted to do so and specific screens with the questions will be visible in which to make responses. The activity tracker and your cell phone will look and work in the manner of a usual and customary tracker and phone. You will not be identifiable or stand out as someone who is using an unusual phone/tracker.
5. All questions will be asked in a professional and courteous manner. Should any of the questions cause you any discomfort you may decline to answer them; this includes during initial/final assessments and any survey or phone-based self-report question. If you decline to answer some questions, the interviewer may ask additional and related questions to determine if there is any risk to your safety.
6. We will never capture information from phone calls or text messages, such as content or specific text.

As with any research study, there may be additional risks that are unknown or unexpected, including the risk of using a cell phone. The use of a cell phone while driving is prohibited in many states including Michigan. You will be expected to abide by the laws governing the use of cellular telephones at all times. Should you choose to use the phone in a manner that is not legal (e.g., while driving) you are solely responsible for the consequences.

### 5.2 What happens if I get hurt, become sick, or have other problems as a result of this research?

The researchers have taken steps to minimize the risks of this study. Even so, you may still have problems or side effects, even when the researchers are careful to avoid them. Please tell the researchers listed in Section 10 about any injuries, side effects, or other problems that you have during this study. You should also tell your regular doctors.

**5.3 If I take part in this study, can I also participate in other studies?**

*Being in more than one research study at the same time, or even at different times, may increase the risks to you. It may also affect the results of the studies*. You should not take part in more than one study without approval from the researchers involved in each study.

**5.4 How could I benefit if I take part in this study? How could others benefit?**

You may not receive any personal benefits from being in this study. However, some subjects may see improvement in their mood-related symptoms. Potential benefits of the research for society and mankind include a better understanding of how to help individuals with bipolar disorder monitor and maintain their symptoms on a regular basis.

**5.5 Will the researchers tell me if they learn of new information that could change my willingness to stay in this study?**

Yes, the researchers will tell you if they learn of important new information that may change your willingness to stay in this study. If new information is provided to you after you have joined the study, it is possible that you may be asked to sign a new consent form that includes the new information.

## 6. OTHER OPTIONS

**6.1 If I decide not to take part in this study, what other options do I have?**

Participation is entirely voluntary and any treatment you may need will not be affected in any way by choosing not to participate in this study. There may be other ways to treat bipolar disorder, including treatment with medication, alternative treatments such as psychosocial therapy, or other experimental treatments. Your doctor can provide you more information about these other treatments, their risks and their possible benefits. You should have this discussion about the risks and benefits or other alternatives prior to making your decision about whether or not you wish to take part in this study.

## 7. ENDING THE STUDY

**7.1 If I want to stop participating in the study, what should I do?**

You are free to leave the study at any time. If you leave the study before it is finished, there will be no penalty to you. You will not lose any benefits to which you may otherwise be entitled. If you choose to tell the researchers why you are leaving the study, your reasons for leaving may be kept as part of the study record. If you decide to leave the study before it is finished, please tell one of the persons listed in Section 10 “Contact Information” (below).

**7.2 Could there be any harm to me if I decide to leave the study before it is finished?**

You may withdraw from this study at any time. There are no penalties for doing so and withdrawing will not pose any dangers to you.

**7.3 Could the researchers take me out of the study even if I want to continue to participate?**

Yes. There are many reasons why the researchers may need to end your participation in the study. Some examples are:

- ✓ The researcher believes that it is not in your best interest to stay in the study.
- ✓ You become ineligible to participate.
- ✓ Your condition changes and you need treatment that is not allowed while you are taking part in the study.
- ✓ You do not follow instructions from the researchers.
- ✓ The study is suspended or canceled.

## 8. FINANCIAL INFORMATION

### 8.1 Who will pay for the costs of the study? Will I or my health plan be billed for any costs of the study?

The study will pay for research-related items or services that are provided only because you are in the study. If you are not sure what these are, see Section 4.1 above or ask the researchers for a list. If you get a bill you think is wrong, call the researchers' number listed in section 10.1.

You or your health plan will pay for all the things you would have paid for even if you were not in the study, like:

- Health care given during the study as part of your regular care
- Items or services needed to give you study drugs or devices
- Monitoring for side effects or other problems
- Deductibles or co-pays for these items or services.

If you do not have a health plan, or if you think your health plan may not cover these costs during the study, please talk to the researchers listed in Section 10 below or call your health plan's medical reviewer.

By signing this form, you do not give up your right to seek payment if you are harmed as a result of being in this study.

### 8.2 Will I be paid or given anything for taking part in this study?

There is potential for you to earn up to a total of $190 dollars for participating in this study. You will be paid $45 for completion of the initial interview. You will be paid an additional $20 for each weekly interview. There is a maximum of five weekly interviews, so you can earn up to $100 (=$20 x 5) for these weekly interviews. Lastly, you will be paid an additional $45 for the final interview. If you choose to leave the study early, you will still be paid for each completed assessment up to your date of termination and for returning the study equipment. If the equipment is not returned, your final payment compensating you for participation may be forfeited. All payments will be distributed by a check sent directly to your home. If you receive any payments for taking part in the study, the University of Michigan accounting department will collect your name, address, social security number, payment amount, and related information.

### 8.3 Who could profit or financially benefit from the study results?

No person or organization has a financial interest in the outcome of the study.

## 9. CONFIDENTIALITY OF SUBJECT RECORDS AND AUTHORIZATION TO RELEASE YOUR PROTECTED HEALTH INFORMATION

The information below describes how your privacy and the confidentiality of your research records will be protected in this study.

### 9.1 How will the researchers protect my privacy?

Study records that contain patient names will have access limited to the Principal Investigator and the immediate study team that is collecting the data. Confidentiality will be preserved by coding all study data with a unique identifying research number, and referring to this number in all analyses. You will not be identified in any reports on this study. Personally-identifiable information such as your name and email address will be stored using a secure, HIPAA-compliant, web-based application known as REDCap. Data from REDCap will be downloaded through secure internet connections and stored in secure, restricted access servers which are behind a firewall, under intrusion monitoring, and will be made accessible only to the members of the study team. Data downloaded from REDCap will have all personally-identifiable information (e.g., participants' names, email addresses) removed and will be coded with a participant ID. Coded data will be stored separately from the master link between the participant's personally-identifiable information and participant ID. Each data set will be kept

secure and confidential in the manner required by federal, state, and local law. We will do everything we can to keep others from learning about your participation in the research.

**9.2 What information about me could be seen by the researchers or by other people? Why? Who might see it?**

Signing this form gives the researchers your permission to obtain, use, and share information about you for this study, and is required in order for you to take part in the study. Information about you may be obtained from any hospital, doctor, and other health care provider involved in your care, including:

- Hospital/doctor's office records, including test results (X-rays, blood tests, urine tests, etc.)
- Mental health care records (except psychotherapy notes not kept with your medical records)

There are many reasons why information about you may be used or seen by the researchers or others during or after this study. Examples include:

- The researchers may need the information to make sure you can take part in the study.
- The researchers may need the information to check your test results or look for side effects.
- University, Food and Drug Administration (FDA) and/or other government officials, auditors, and/or the IRB may need the information to make sure that the study is done in a safe and proper manner.
- Study sponsors or funders, or safety monitors or committees, may need the information to:
    - Make sure the study is done safely and properly
    - Learn more about side effects
    - Analyze the results of the study
- Insurance companies or other organizations may need the information in order to pay your medical bills or other costs of your participation in the study.
- The researchers may need to use the information to create a databank of information about your condition or its treatment.
- Information about your study participation may be included in your regular UMHS medical record.
- If you receive any payments for taking part in this study, the University of Michigan accounting department may need your name, address, social security number, payment amount, and related information for tax reporting purposes.
- Federal or State law may require the study team to give information to government agencies. For example, to prevent harm to you or others, or for public health reasons.

The results of this study could be published in an article, but would not include any information that would let others know who you are.

A description of this clinical trial will be available on http://www.clinicaltrials.gov/, as required by U.S. Law. This Web site will not include information that can identify you. At most, the Web site will include a summary of the results. You can search this Web site at any time.

**9.3 What happens to information about me after the study is over or if I cancel my permission?**

As a rule, the researchers will not continue to use or disclose information about you, but will keep it secure until it is destroyed. Sometimes, it may be necessary for information about you to continue to be used or disclosed, even after you have canceled your permission or the study is over.

Examples of reasons for this include:

- To avoid losing study results that have already included your information
- To provide limited information for research, education, or other activities (This information would not include your name, social security number, or anything else that could let others know who you are.)
- To help University and government officials make sure that the study was conducted properly

As long as your information is kept within the University of Michigan Health System, it is protected by the Health System's privacy policies. For more information about these policies, ask for a copy of the University of Michigan "Notice of Privacy Practices". This information is also available on the web at http://www.uofmhealth.org/patient+and+visitor+guide/hipaa. Note that once your information has been shared with others as described under Question 9.2, it may no longer be protected by the privacy regulations of the federal Health Insurance Portability and Accountability Act of 1996 (HIPAA).

**9.4 When does my permission expire?**

Your permission expires at the end of the study, unless you cancel it sooner. You may cancel your permission at any time by writing to the researchers listed in Section 10 "Contact Information" (below).

## 10. CONTACT INFORMATION

**10.1 Who can I contact about this study?**

Please contact the researchers listed below to:

- Obtain more information about the study
- Ask a question about the study procedures or treatments
- Talk about study-related costs to you or your health plan
- Report an illness, injury, or other problem (you may also need to tell your regular doctors)
- Leave the study before it is finished
- Express a concern about the study

**Principal Investigator:** Melvin McInnis, MD
**Mailing Address:**
Heinz C. Prechter Bipolar Research Fund
University of Michigan Depression Center
Rachel Upjohn Building
4250 Plymouth Road
Ann Arbor, MI 48109-2700
**Telephone:** 1-877-864-3637

You may also express a concern about a study by contacting the Institutional Review Board listed below.

University of Michigan Medical School Institutional Review Board (IRBMED)
2800 Plymouth Road
Building 520, Room 3214
Ann Arbor, MI 48109-2800
Telephone: 734-763-4768 (For International Studies: US Country Code: 001)
Fax: 734-763-1234
e-mail: irbmed@umich.edu

If you are concerned about a possible violation of your privacy or concerned about a study you may contact the University of Michigan Health System Compliance Help Line at 1-866-990-0111.

*When you call or write about a concern, please provide as much information as possible, including the name of the researcher, the IRBMED number (at the top of this form), and details about the problem. This will help University officials to look into your concern. When reporting a concern, you do not have to give your name unless you want to.*

## 11. RECORD OF INFORMATION PROVIDED

### 11.1 What documents will be given to me?

Your signature in the next section means that you have received copies of all of the following documents:

- A copy of the signed and dated "Consent to be Part of a Research Study" document. (*Note: In addition to the copy you receive, copies of this document will be stored in a separate confidential research file and may be entered into your regular University of Michigan medical record.)*

## 12. SIGNATURES

**Consent/Assent to Participate in the Research Study**

I understand the information printed on this form. I have discussed this study, its risks and potential benefits, and my other choices with __________________________. My questions so far have been answered. I understand that if I have more questions or concerns about the study or my participation as a research subject, I may contact one of the people listed in Section 10 (above). I understand that I will receive a copy of this form at the time I sign it and later upon request. I understand that if my ability to consent or assent for myself changes, either I or my legal representative may be asked to re-consent prior to my continued participation in this study.

Legal Name: ____________________________________________________________

Signature: ____________________________________________________________

Date of Signature (mm/dd/yy): __________________________

**Principal Investigator or Designee**

I have provided this participant and/or his/her legally authorized representative(s) with information about this study that I believe to be accurate and complete. The participant and/or his/her legally authorized representative(s) indicated that he or she understands the nature of the study, including risks and benefits of participating.

Legal Name: ____________________________________________________________

Title: ________________________________________________________________

Signature: ____________________________________________________________

Date of Signature (mm/dd/yy): __________________________
