# Supplementary material for: Engagement Strategies for Self-Monitoring Symptoms of Bipolar Disorder With Mobile and Wearable Technology: Protocol for a Randomized Controlled Trial
Source: JMIR Res Protoc. 2018 May 10;7(5):e130. doi: 10.2196/resprot.9899 (PMC5968216; doi:10.2196/resprot.9899)
Supplement: Multimedia Appendix 2 [file resprot_v7i5e130_app2.pdf]

Protocol for Aim 2

Modeling Mood Course to Detect Markers for Effective Adaptive Interventions

**Date:**	11/2/17
**Version:**	2
**Principal Investigator:**	Melvin McInnis
**Contact:**	Heinz C. Prechter Bipolar Research Fund<br>University of Michigan Depression Center<br>Rachel Upjohn Building<br>4250 Plymouth Road<br>Ann Arbor, MI 48109-2700<br>bpresearch@umich.edu<br>1-877-864-3637
**Institute:**	University of Michigan

Contents

Introduction....3

A. Type of Research....3

B. Purpose/Objective of the Study....3

C. Background of the Study....3

Participant Selection....4

A. Inclusion and exclusion criteria....4

B. Gender....4

C. Racial/Ethnic Origin....4

D. Vulnerable Populations....4

E. Age....5

F. Total Number of Participants to be Enrolled....5

Study Design / Method / Procedures....5

A. Summary of the Research Design....5

B. Analysis of Study Results....7

C. Monitoring....8

D. Storage and Confidentiality of Data....8

Participant Recruitment and Informed Consent....8

A. Recruiting....8

B. Informed Consent / Assent....8

Introduction

A. Type of Research

The research to be conducted under this protocol falls under the general heading of a clinical trial with a behavioral intervention. It investigates how individuals with bipolar disorder interact with mobile and wearable technology, incorporating both qualitative and quantitative analyses.

B. Purpose/Objective of the Study

Our long-term objective is to develop a mobile health platform for the translation of a psychosocial intervention for bipolar disorder (BP) into an effective adaptive intervention. In this particular part of the study, we explore how to engage individuals with BP in long-term monitoring of their daily patterns of mood, stress, sleep, circadian rhythm, and medical adherence. To answer this question, we will study how individuals with BP interact with a smart-phone application and activity tracker. Three hypotheses will be tested for how to engage individuals with BP: individuals with BP prefer and better adhere to monitoring of their daily patterns when...

(H1) using activity trackers over self-reports;
(H2) they review weekly symptoms with another person; and
(H3) the collected data is synthesized into information perceived to offer new insight.

C. Background of the Study

Bipolar (BP) disorder is a chronic illness of profound shifts in mood ranging from mania to depression. BP is successfully treated by combining medication with psychosocial therapy, but care can prove inadequate in practice. With gaps in coverage and medication, along with imprecise guidelines on when, where, and how to intervene, promising psychosocial therapies require adaptive strategies to better address the specific needs of individuals in a timely manner (NIMH Strategy 2). To accomplish this, however, requires evidence-based practices for adapting a psychosocial therapy. This project aims to address this knowledge gap by establishing a mobile health platform for translating a psychosocial therapy in BP into an effective adaptive intervention.

To adapt a psychosocial therapy to information in the moment, then symptoms should be monitored frequently over extended periods. Frequent observations (multiple times a day) are needed to capture the diurnal patterns (Wirz-Justice 2008) and rapid mood shifts (Kramlinger and Post 1996) associated with BP. Extended observation (weeks to years) captures full length episodes (Solomon, Leon et al. 2010) and certain medication effects (Goodwin, Jamison et al. 2007).

Mood is commonly charted with self-reports, often on a one-dimensional scale such as the Mood 24/7 scale (April C. Foreman 2011) and the NIMH Life Chart Method (Denicoff, Leverich et al. 2000). However, mood in BP may not be one-dimensional. NIMH's RDoC, for example, defines both a negative valence and positive valence domain (Cuthbert and Insel 2013). Accuracy of self-reports may also depend on an individual's mood (Born, Amann et al. 2014) and time of day (Wirz-Justice 2008). They also require individuals to *actively* record symptoms, a burden that leads to disengagement (Nahum-Shani, Smith et al. 2014). In sum, self-reports are subjective and time-intensive and lose information that limit its utility in charting BP.

BP can also be charted *passively* through sensors on smart-phones and wearable devices (Depp, Mausbach et al. 2010). Smart-phone platforms MONARCA (MONitoring, treAtment and pRediCtion of bipolAr Disorder Episodes) and PRIORI (Predicting Individual Outcomes for Rapid Intervention; McInnis, PI, R34MH100404) aim to predict mood from patterns of speech and behavior from recorded calls, number of phone calls, and phone call duration. However, smart-phones do not monitor circadian and sleep rhythms, a primary target of therapies such as sleep deprivation (Wu, Kelsoe et al. 2009) and interpersonal and social rhythm therapy (Frank, Kupfer et al. 2005). Disrupted circadian rhythms are thought to be central to BP etiology, with connections to risk genes, animal models, and pharmacological therapy (McClung 2013).

Actigraphy is a promising alternative that can evaluate sleep and circadian rhythms. Pagani, Clair et al. (2015) have linked actigraphic variables to genetic differences between BP-I and non-BP-I individuals that include later wake times, longer sleep durations, and lower activity levels in BP-I subjects during euthymia. In a current study with the investigators, actigraphy data revealed that BP individuals wake up significantly later than non BP individuals, while reporting they are less rested, drink more caffeine, and use more sleep

medication. Lower activity is also found to be significantly associated with an increase in depressive symptoms, whereas higher activity is associated with an increase in manic symptoms. A validated mathematical model of circadian rhythms were developed by Dr. Daniel Forger (Forger, Jewett et al. 1999). With these models and statistics, Dr. Forger, his student Olivia Walch, and a co-investigator of the current study, Amy Cochran, analyzed sleep habits from over 5000 users from over 100 countries collected with a smartphone app ENTRAIN (Walch, Cochran et al. 2016). This work has been published in Science Advances and featured in over 100 media outlets across the globe that include Huffington Post, BBC, Wired, and TIME.

Participant Selection

A. Inclusion and exclusion criteria

We will recruit 54 individuals from the Prechter Longitudinal study of Bipolar Disorder who (i) have agreed to be contacted for future research, (ii) have a smart-phone, and (iii) have either a diagnosis of bipolar disorder or do not have a diagnosis of bipolar disorder. Four individuals without a diagnosis of bipolar disorder will be used to test instruments for two-week study. Once instruments are tested, 50 individuals with a diagnosis of bipolar disorder will be recruited for a six-week study.

B. Gender

Participation is open to men and women. For the six-week study, we will recruit 25 women and 25 men to balance gender in the 50 individuals.

C. Racial/Ethnic Origin

Participation is open to all ethnic and racial groups. The research team's capacity to enroll a study sample with adequate representation of minorities is enhanced by the diversity of Washtenaw and surrounding Counties, in which the majority of the clinic patients live. According to the U.S. Census' American Fact Finder 2010 Demographic Profile Data, the demographics of the population of Washtenaw County are estimated as follows:

- 72.1% White alone, not Hispanic or Latino
- 12.7% Black or African American
- 7.9% Asian
- 4.0% Latino or Hispanic
- 3.4% Two or more races
- 0.3% American Indian or Alaskan Native
- 0.0% Native Hawaiian or Other Pacific Islander

Recruitment milestones have been submitted to the National Institute of Health (see table below). Actual recruitment will be reported to the NIH every four months.

	Total	Racial Minority	Hispanic
**August 1, 2017**	0	0	0
**December 1, 2017**	10	2	1
**April 1, 2018**	22	5	2
**August 1, 2018**	35	9	3
**December 1, 2018**	50	13	4

D. Vulnerable Populations

The study is limited to a subgroup of the population which does not specifically target pregnant women and/or fetuses; lactating women; women of child-bearing potential; prisoners; cognitively impaired adults; college students; economically or educationally disadvantaged persons; patients of the study team; employees, students or trainees of the study team; or family members of the study team.

E. Age

Participation is open to adults (18 years of age or older). For the six-week study, we will recruit 24 individuals under the age of 40 and 26 individuals 40 years of age or older, with equal numbers of women and men in each age group.

F. Total Number of Participants to be Enrolled

A total of 54 individuals will participate in either the two-week study or the six-week study.

Study Design / Method / Procedures

A. Summary of the Research Design

Recruitment and Arms. Fifty individuals with BP will be recruited from the Prechter Longitudinal Study of Bipolar Disorder (PI: Melvin McInnis; HUM606) to participate in a six-week study. They have already completed a Diagnostic Interview for Genetic Studies (DIGS) about their health and mental illness history. We will access the diagnostic information and other data from the longitudinal study so not to repeat the interview process. Each subject in the six-week study. will also be randomly assigned to one of two arms (Arm NR=No Review or Arm R=Review), stratified by age and gender. Four individuals without BP will also be recruited from the Prechter study to pilot the instruments over two weeks; these four individuals will all be assigned to Arm R. The study is necessarily not blinded.

Randomization. Prior to the start of the study, a randomized list of assignment to one of the two arms will be generated for each of four groups: women < 40 years of age (N=12), women ≥ 40 years of age (N=13), men < 40 years of age (N=12), men ≥ 40 years of age (N=13). For the two groups of 12 participants, the randomized list will be comprised of two random blocks of size four and two random blocks of size two with blocks placed in random order. For the two groups of 13 participants, the randomized list will be comprised of two random blocks of size four, one random block of size three, and one random block of size two with random blocks in placed in random order. Together, there will be two random blocks of size three. Because the size is an odd number, these blocks will each be unbalanced, but together will ensure equal participants in each arm; which arm is over-represented in each group is chosen uniformly at random. Using logical statements and subject ids, randomization will be automatically embedded as a computed variable in REDCap.

Entrance interview. ***A summary of participation in the study is given in the table below.*** First, we will mail each participant an activity tracker like a FitBit or an Apple Watch, along with a return box with postage paid. Once they receive the activity tracker, we will schedule a time to talk to them over the phone to assess their mood and functioning and to explain how to set-up and use the study smartphone application and activity tracker, including how to report and review your information and set preferences for notifications.

Activity tracker and smart-phone application. We will ask each participant to wear the activity tracker at all times, except to charge the tracker or to shower. Automatic messages will be sent to the participant to remind them to wear the tracker. The tracker will automatically collect information about physical activity and sleep. The smart-phone application will automatically prompt the user twice-a-day (once in the morning and once in the evening) on questions about their mood, stress, bed and wake times, and medication adherence. These questions will be prompted automatically by the smart-phone app. The smart-phone app will also allow the participant to review their information provided to the study team, including information collected from the activity tracker and processed offline by computational algorithms.

Weekly interviews. We will also contact each participant weekly to complete questionnaires to assess the severity of depressive symptoms and manic symptoms. In addition to these assessments, the participants in Arm R will also review with the study team what you reported over the week about your mood, stress, and medication adherence, and what the activity tracker reported about their sleep and physical activity.

Exit interview. At the end of the study, we will interview each participant to assess functioning, mood, and engagement/preference with smart-phone application and activity tracker. This assessment will take about one hour. The participant will then be asked to mail any study equipment (e.g., activity tracker) using the return box.

Table 1. Summary of participation in study.

Name	Instruments	Time per event (min)	Event time
**Entrance Phone Interview** ***Investigator provided data***	• Young Mania Rating Scale (YMRS)<br>• Structured Interview Guide for the Hamilton Depression Rating Scale (SIGH-D)<br>• 36-Item Short Form Survey (SF-36)<br>• Set-up<br>o Subject ID<br>o Prechter ID<br>o Typical wake time and bed time<br>o Weekly interview time<br>o Medication type, time, and frequency	60	Day 0
**Reminder** ***Automated***	• Message reminder to wear activity tracker	0	Days 1-42;<br>Early morning<br>Early morning = 2 hours after subjects "typical" wake time
**Daily Self-Report through App** ***User provided data***	• Brief inventory of symptoms and medication adherence:<br>o Shortened YMRS<br>o Shortened SIGH-D<br>o Stress<br>o Bed and wake times (morning only)<br>o Medication adherence (at most once daily; depends on medication routine)	5	Days 1-42;<br>Morning and Evening<br>Morning (Evening) = first (last) quarter of subjects "typical" wake period
**Activity Tracker** ***Automated***	• Uploaded daily activity data	0	Days 1-42;<br>Mid-Night<br>Mid-Night = middle of subjects "typical" sleep period
**Daily Computed Variables** ***Automated***	• Computed variables:<br>o Circadian phase<br>o Physical and sedentary behavior<br>o Sleep duration<br>o Longitudinal patterns of sleep, mood, and stress	0	Days 1-42;<br>Mid-Night
**Weekly Phone Interview** ***Investigator provided data***	• YMRS<br>• SIGH-D<br>• Review (Arm R only) of collected and computed variables through activity tracker	Arm NR: 20<br>Arm R: 40	Days 7, 14, 21, 28, 35;<br>Weekly meeting time
**Exit Phone Interview** ***Investigator provided data***	• YMRS<br>• SIGH-D<br>• SF-36<br>• Review (Arm R only)<br>• Engagement with monitoring of symptoms:<br>o Preference and burden: self-report<br>o Preference and burden: activity tracker<br>o Preference and burden: computed variables<br>o Preference and burden: weekly interviews (+review)<br>o Comparison: self-report vs. activity tracker<br>o Comparison: computed vs. non-computed variables	60	Day 42;<br>Weekly meeting time

Study termination. The study team may decide to end participation of any person in the study if (i) the team believes it is not in the person's best interest to stay in the study; (ii) the person becomes ineligible; (iii) the person's condition changes or needs treatment that is not allowed while taking part in the study; (iv) the person does not follow instructions from the study team; or (v) the study has been suspended or cancelled.

Suicidality. We will ask about questions about suicide (e.g., attempts, ideation) as part of the Structured Interview Guide for the Hamilton Rating Scale for Depression. The outpatient psychiatry department at the University of Michigan has standard procedures that are followed when there is a concern for patient safety and the University of Michigan psychiatric emergency room is available 24/7. In instances when the study team anticipates a threat of self-harm, or harm to others due to the individuals worsening psychiatric condition, we will take all clinically indicated appropriate actions to protect the individual, which may include hospitalization. We may need to breach confidentiality in these instances, but will limit the sharing of knowledge to the bare minimum required for safety.

B. Analysis of Study Results

Our primary outcomes evaluate three hypotheses about how to engage individuals with BP, which are that individuals with BP prefer and better adhere to monitoring of their daily patterns when...

(H1) using activity trackers over self-reports;
(H2) they review weekly symptoms with another person; and
(H3) the collected data is synthesized into information perceived to offer new insight.

To evaluate these hypotheses, we measure ***adherence to activity trackers*** in each individual as the proportion of days with at least 16 hours of wearing the activity track and ***adherence to self-reports*** in each individual as the proportion of days with at least 2/3 of the daily self-report questions answered. Note that 16 hours is 2/3 of a day and is chosen to correspond with the 2/3 of daily questions used to defined adherence to self-reports. Here, a day is considered to start and end at 12am. For (H1), we use two outcome measures:

- Proportion of individuals with preference for activity trackers over self-reports to monitor their daily patterns, as reported at the end of the study, and
- Average difference in adherence to activity trackers and adherence to self-reports.

For (H2), we use three outcome measures: difference between Arm R and Arm NR in the

- Average adherence to activity trackers,
- Average adherence to self-reports, and
- Proportion of individuals with a preference for monitoring their daily patterns over not monitoring, as reported at the end of the study.

For (H3), we use two outcome measures: difference between individuals who report computed variables (e.g, circadian phase) did offer new insight into their daily patterns and those individuals who report that the computation variables did not offer new insight in the

- Average adherence to activity tracker, and
- Average adherence to self-report.

Our secondary outcomes focus on understanding what factors impact the change from baseline to 6 weeks in SF-36, YMRS, and SIGHD scores. Secondary outcomes of particular interest are

- Average difference in changes in SF-36, YMRS, and SIGHD scores between Arm R and Arm NR
- Cross-correlation coefficients between
    - Average adherence to activity trackers and changes in SF-36, YMRS, and SIGHD scores
    - Average adherence to self-reports and changes in SF-36, YMRS, and SIGHD scores
- Average difference in changes in SF-36, YMRS, and SIGHD scores between individuals that report at the end of the study a preference for monitoring daily symptoms over not monitoring symptoms

To analyze these outcome measures, we will use a one-way ANOVA to measure differences in covariates between groups and cross-tabulation with a Pearson's chi-squared test to measure differences in categorical variables between groups. Significance will be considered an alpha level of 0.05.

C. Monitoring

Monitoring safety of the study will be the responsibility of the research team and principal investigator. On a monthly basis, the candidate with a research assistant of the Prechter study will review any serious adverse events; drop-outs and non-serious adverse events; proposed amendments to the study protocol including those for reasons related to the safety of study subjects; completeness and validity of data to be used for analyses; progress towards Recruitment Milestone Report (RMR) for all subjects and for minority recruitment; and any violation/deviations in protocol including breach in subject privacy and confidentiality. Immediately upon review, reports will be relayed to the Prechter study team and Dr. Melvin McInnis and any adverse events including violation/deviations in protocol will be reported to the IRBs. Each adverse event will also be assessed according to its relatedness to the proposed study and expectedness as specified by the IRB, informed consent, and protocols. As in any research study, it is not possible to anticipate all possible adverse events. The Prechter team has substantial experience in monitoring, interviewing, and treating individuals with bipolar disorder. They do extensive training with our staff on ascertaining, monitoring, and documenting adverse events. Established procedures are in place at the site for rendering first aid and life threatening emergencies. In the case of this study, all investigators and study staff will be trained in monitoring and documenting adverse events.

D. Storage and Confidentiality of Data

We will the secure, HIPAA-compliant web-based application known as REDCap, as well as fully automated encryption technology and secure websites (https) for data capture. Study records that contain patient names will have access limited to the Principal Investigator and the immediate study team that is collecting the data. Confidentiality will be preserved by coding all study data with a unique identifying research number, and referring to this number in all analyses. Data from REDCap will be downloaded through secure internet connections and stored in secure, restricted access servers which are behind a firewall, under intrusion monitoring; will be made accessible only to the members of the study team; and will have all personally-identifiable information (e.g., participants' names, email addresses) removed and will be coded with a participant ID. Data will be backed up regularly on secure, restricted access servers that are administered by the University of Michigan School of Information's highly trained information technology department. Coded data will be stored separately from the link between the participant's personally-identifiable information and participant ID. In addition, the smart-phone application will use fully automated encryption technology and secure websites for data capture to minimize the risk of security breach to the files.

Participant Recruitment and Informed Consent

A. Recruiting

Participants will be recruited through email or phone from the Prechter Longitudinal Study of Bipolar Disorder (HUM606) who have agreed to be contacted for future studies.

B. Informed Consent / Assent

The following steps will be taken to consent participants for the study:

1) Participants will be emailed a pdf version of the consent form prior to the start of the study. The form will provide important information about the study, including the purpose of the study, and the risks and possible benefits of participating in the study.
2) A member of the study team will phone each participant, provide a complete description of the study to the participants, answer any questions, and give them enough time to participate.
3) At the end of the phone call, the participant will be emailed from REDCap an electronic version of the consent form that is designed as a REDCap survey.
4) If the participant decides to participate, the electronic version can be completed and will be identical to the pdf version of the consent form in content and order. Each of the 12 sections of the consent form will be contained on separate pages of the electronic consent that the participant will click through. At the 12th section, the participant will be asked to provide an electronic version of their signature through the use of a mouse or touch-screen device and then submit the consent form.

5) The study member receiving the consent form will print the signed consent form and then sign and date the form. Each participant will be emailed a copy of his or her signed informed consent form and the printed form will be placed in a secure filing location within a locked cabinet behind locked doors.
