# Supplementary material for: Engagement Strategies for Self-Monitoring Symptoms of Bipolar Disorder With Mobile and Wearable Technology: Protocol for a Randomized Controlled Trial
Source: JMIR Res Protoc. 2018 May 10;7(5):e130. doi: 10.2196/resprot.9899 (PMC5968216; doi:10.2196/resprot.9899)
Supplement: Multimedia Appendix 3 [file resprot_v7i5e130_app3.pdf]

Engagement Assessment

Record ID ______________________

1. How much did you learn from using a smart-phone app and an activity tracker to monitor your mood?

- ◯ I learned nothing
- ◯ I learned a little
- ◯ I learned a fair bit
- ◯ I learned a lot

2. How much do you feel your emotional health improved by using a smart-phone app and an activity tracker to monitor your mood?

- ◯ It did not improve
- ◯ Improved a little
- ◯ Improved a fair amount
- ◯ Improved a lot

---

3. Each question below is about monitoring symptoms with either an activity tracker (such as a Fitbit) or a smart-phone app (such as the one provided in this study).

	An activity tracker	A smart-phone app
3a. Which are you more likely to use to monitor your symptoms?	◯	◯
3b. Which did more to improve your emotional health?	◯	◯
3c. Which required less effort to use?	◯	◯
3d. Which was less complicated to use?	◯	◯
3e. Which did you learn more from?	◯	◯

The following questions are about monitoring your symptoms with a smart-phone app.

4. How long would you continue to monitor your symptoms with a smart-phone app?

- ◯ Less than a month
- ◯ 1-6 months
- ◯ 6-12 months
- ◯ Over a year

5. How much effort was required to use a smart-phone app to monitor your symptoms?

- ◯ No effort
- ◯ Little effort
- ◯ A fair amount of effort
- ◯ A lot of effort

6. How complicated was it to use a smart-phone app to monitor your symptoms?

- ◯ Not complicated at all
- ◯ Slightly complicated
- ◯ Fairly complicated
- ◯ Very complicated

The following questions are about monitoring your symptoms with an activity tracker.

7. How long would you continue to monitor your symptoms with an activity tracker?
- ◯ Less than a month
- ◯ 1-6 months
- ◯ 6-12 months
- ◯ Over a year

8. How much effort was required to use an activity tracker to monitor your symptoms?
- ◯ No effort
- ◯ Little effort
- ◯ A fair amount of effort
- ◯ A lot of effort

9. How complicated was it to use an activity tracker to monitor your symptoms?
- ◯ Not complicated at all
- ◯ Slightly complicated
- ◯ Fairly complicated
- ◯ Very complicated

The following questions are about visualizing your daily symptoms and patterns with charts or graphs.

10. How long would you continue to visualize your daily symptoms and patterns with charts?
- ◯ Less than a month
- ◯ 1-6 months
- ◯ 6-12 months
- ◯ Over a year

11. How much did you learn from visualizing your daily symptoms and patterns with charts?
- ◯ I learned nothing
- ◯ I learned a little
- ◯ I learned a fair amount
- ◯ I learned a lot

12. How complicated was it to visualize your daily symptoms and patterns with charts?
- ◯ Not complicated at all
- ◯ Slightly complicated
- ◯ Fairly complicated
- ◯ Very complicated

The following questions are about discussing symptoms recorded by a smart-phone app or an activity tracker with another person.

13. How frequently would you want to review your recorded symptoms with another person?
- ◯ Never
- ◯ Once a year
- ◯ Monthly
- ◯ Weekly

14. Who would you prefer to review your symptoms with?
- ◯ A clinician (such as a doctor, nurse, or psychologist)
- ◯ A family member
- ◯ A friend
- ◯ Someone I did not know and who did not know me
- ◯ Someone who also has bipolar disorder

The remaining questions are about monitoring your symptoms with technology in general.

15. What are the top three symptoms or patterns for you to monitor?

	1	2	3
Sleep	◯	◯	◯
Stress	◯	◯	◯
Mood	◯	◯	◯
Energy	◯	◯	◯
Irritability	◯	◯	◯
Regularity in schedules/routines	◯	◯	◯
Anxiety	◯	◯	◯
Physical activity	◯	◯	◯
Heartrate	◯	◯	◯
Medication adherence	◯	◯	◯

16. What do you consider to be the top three uses of technology for monitoring your symptoms?

	1	2	3
Improve symptoms	◯	◯	◯
Change behavior and habits	◯	◯	◯
Maintain a regular schedule or routine	◯	◯	◯
Encourage physical exercise	◯	◯	◯
Raise self-awareness of symptoms	◯	◯	◯
Predict an upcoming episode	◯	◯	◯
Alert someone when I am having trouble	◯	◯	◯
Remind me to take my medicine	◯	◯	◯
Learn something about my health	◯	◯	◯
Provide information to my clinicians	◯	◯	◯

17. What do you consider to be the top three barriers preventing you from using technology to monitor your symptoms?

	1	2	3
Unclear improvement in my health	○	○	○
No one keeps me accountable	○	○	○
Costs too much	○	○	○
Too complicated to use	○	○	○
Requires too much effort	○	○	○
Concerned about my privacy and/or data security	○	○	○
Not recommended by my doctor	○	○	○
Don't know which device or app to use	○	○	○
Forgetfulness	○	○	○
Not engaging enough (e.g., boring)	○	○	○
